# Complete Genome Sequence of Escherichia coli Sequence Type 1193 Isolate AVS0096, Recovered from River Water in Switzerland

**DOI:** 10.1128/MRA.00607-21

**Published:** 2021-08-05

**Authors:** Michael Biggel, Sarah Hoehn, Kira Schmitt, Andrea Frei, Christoph Jans, Roger Stephan

**Affiliations:** a Institute for Food Safety and Hygiene, Vetsuisse Faculty University of Zürich, Zürich, Switzerland; b Amt für Verbraucherschutz, Kanton Zug, Steinhausen, Switzerland; Indiana University, Bloomington

## Abstract

Escherichia coli sequence type 1193 (ST1193) is an important cause of multidrug-resistant extraintestinal infections. Here, we report the complete genome sequence of strain AVS0096, isolated from river water in Switzerland in 2020. The genome consists of a chromosome (4.9 Mbp), a multidrug resistance plasmid (101 kb), and two small plasmids.

## ANNOUNCEMENT

The pandemic Escherichia coli lineages sequence type 131-*H*30 (ST131-*H*30) and ST1193 are major causative agents of fluoroquinolone-resistant E. coli infections in humans (1). Both lineages have acquired chromosomal mutations in the quinolone resistance-determining regions (QRDRs) of *gyrA* and *parC* and frequently carry additional resistance determinants such as extended-spectrum beta-lactamases (ESBLs) (2–6). Whereas the evolution and gene content of ST131 have been investigated in depth (5, 6), few studies have addressed ST1193, and complete genome assemblies of ST1193 are limited. Here, we report the complete genome sequence of a *bla*_CTX-M-27_-carrying ST1193 isolate (AVS0096) obtained from river water in Switzerland.

AVS0096 was isolated in August 2020 from the river Lorze. The water sample (100 ml) was filtered through a 0.45-μm membrane filter (Millipore). The filter was incubated in 10 ml *Enterobacteriaceae* enrichment (EE) broth (BD) at 37°C for 24 h. One loopful of the enrichment broth was spread onto Brilliance ESBL agar (Oxoid) and incubated at 37°C for 24 h. Matrix-assisted laser desorption ionization–time of flight mass spectrometry (MALDI-TOF MS; Bruker Daltonics) was used for species identification. Susceptibility testing against 13 antimicrobial agents was performed using the disk diffusion method according to CLSI protocols (7). DNA was isolated from a subculture obtained from a single colony grown for 24 h at 37°C on sheep blood agar. For short-read sequencing, DNA was extracted using the DNeasy blood and tissue kit (Qiagen). Libraries were prepared using the Nextera DNA Flex library preparation kit (Illumina) and sequenced on the Illumina MiniSeq platform (2 × 150 bp). For long-read sequencing, DNA was obtained using the MasterPure complete DNA and RNA purification kit (Lucigen) (no size selection/shearing). Multiplex libraries were prepared using the SQK-LSK109 kit with the EXP-NBD104 barcode kit and sequenced using a MinION device on a FLO-MIN106 flow cell (Oxford Nanopore Technologies). Base calling was performed using Guppy (CPU v4.2.2+effbaf8). Adapters of Illumina and ONT reads were trimmed using Trim Galore v0.6.6 (8) and Porechop v0.2.4 (9) and quality assessed using FastQC v0.11.9 (https://www.bioinformatics.babraham.ac.uk/projects/fastqc/) and LongQC v1.2.0 (10), respectively. A hybrid assembly was generated from 358 Mbp long-read (16,810 reads; *N*_50_, 41 kb; coverage, 72×) and 274 Mbp short-read (922,057 paired-end reads; coverage, 55×) data using Unicycler v0.4.8 (11) and annotated using Prokaryotic Genome Annotation Pipeline (PGAP) v2021-01-11 (12). Resistance genes and plasmid replicons were identified using ABRicate v1.0.1 (13) (coverage/identity, >70%/>90%) with the ResFinder (14) and PlasmidFinder (15) databases, respectively. Default parameters were used for all software unless otherwise specified.

**FIG 1:**
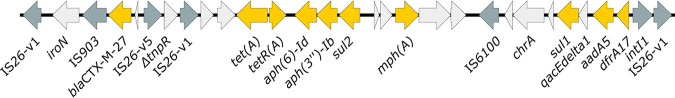
Genetic environment of antimicrobial resistance genes in plasmid pAVS0096-a. The antimicrobial resistance genes and associated insertion elements (IS) and integrases are highlighted.

The complete genome of AVS0096 consisted of a 4,944,762-bp chromosome and the three plasmids pAVS0096-a (101 kb, IncFIA/IncFIB/Col156), pAVS0096-b (44 kb, IncX1), and pAVS0096-c (2 kb, Col[BS512]). AVS0096 belonged to the ST1193-*H*64 clade, as determined using https://pubmlst.org/ and FimTyper v1.0 (16), and carried known QRDR mutations (*gyrA*, S83L and D87N; *parC*, S80I) (2). Nine acquired resistance genes, including the ESBL-encoding *bla*_CTX-M-27_, were found on pAVS0096-a (Fig. 1). A near-identical plasmid (99% query coverage, 99% identity) to pAVS0096-a was identified in the NCBI nucleotide collection, namely, pWP3-W18-CRE-03_1 (GenBank accession number AP021964.1) from E. coli WP3-W18-CRE-03 (ST1193), isolated from wastewater in Japan. According to the CLSI criteria, AVS0096 showed resistance against azithromycin (macrolide), ampicillin, cefazolin, and cefotaxime (β-lactams), ciprofloxacin (fluoroquinolone), streptomycin (aminoglycoside), tetracycline, and trimethoprim-sulfamethoxazole, confirming genotypically identified resistances.

### Data availability.

The complete genome sequence of AVS0096 has been deposited in GenBank under the accession numbers CP076344.1 (chromosome), CP076345.1 (plasmid pAVS0096-a), CP076346.1 (plasmid pAVS0096-b), and CP076347.1 (plasmid pAVS0096-c). The raw data were deposited in the NCBI Sequence Read Archive (SRA) under BioSample accession number SAMN19493560 and BioProject accession number PRJNA734472.
